# Nanometrology

**DOI:** 10.3390/nano12213755

**Published:** 2022-10-26

**Authors:** Petr Klapetek

**Affiliations:** Czech Metrology Institute, Okružní 31, 638 00 Brno, Czech Republic; pklapetek@cmi.cz

Apart from being the subject of this Special Issue, what is nanometrology? The most straightforward definition would be that it is a measurement science related to anything at the nanoscale. This might include various subjects that can be very distinct: measurements of physical and chemical properties with very high spatial resolution, such as in electron microscopy; measurements of properties of various ensembles of nanoscale systems, such as aerosols or nanocomposites; or the development of metrology instrumentation that would be based on some nanoscale phenomena, such as in Surface Enhanced Raman Spectroscopy. Calling any of these disciplines “nanometrology” would make sense, and since this word started to be used in the 1990s, it has been used in very distinct contexts. From the perspective of national metrology institutes (NMI) that have been always very active in the dimensional aspects of measurements at the nanoscale, stimulated by the increasing needs of the semiconductor industry, nanometrology was initially related to providing metrological traceability to dimensional measurements and performing interlaboratory comparisons. In academia, the variety of topics became much wider, focusing on novel measurement principles, sometimes far from any metrological traceability. When looking at some older review papers related to nanometrology, e.g., [1,2], we can see that it was expected that nanometrology could cover nearly all that was mentioned above, expanding also to novel areas such as life sciences. Were these expectations met when we look at the subject more than ten years later?

Starting from the opposite direction, we can determine which subjects have been related to nanometrology in scientific papers. By searching the Web of Science, we can find about 1300 papers related to the word “nanometrology” (searching for all the fields and years) with an average number of citations per publication around 10. Taking the most cited 10% of papers (having more than 25 citations), we can calculate statistics for their topics, materials and methods. Even if we classify the papers manually in order to prevent the largest errors, due to the fact that the relation to word “nanometrology” can be highly variable, the results of this survey are only very rough. However, we can still use the results to obtain a rough idea about how the whole subject has evolved over the years.

Looking from the perspective of metrology institutes, we can see that 30% of papers have at least one author from NMI; 32% are related to metrological traceability and 58% to uncertainties and error sources, but only 12% deal with some kind of methodological comparisons between different instruments, methods or laboratories. The wide range of measurement topics related to optics (including, e.g., optical microscopy, plasmonics or Raman microscopy) is covered by 37% of papers, while the much narrower field of Scanning Probe Microscopy is covered in about the same number, 36% of the papers. Classical dimensional metrology topics, such as building interferometers for providing metrological traceability up to the smallest movement scale, are discussed in 31% of papers. More complex analytical techniques such as X-ray-based surface and thin film analysis are found in only 21% of selected papers. Many of the expected topics have quite low numbers of papers, e.g., methods for thin film analysis are developed in only 6% of the papers (even though many of the other methods can, in principle, be used for thin films as well). There is a lot of variety in the studied materials and sample types, out of which there is one larger group (21%) of papers related to the analysis of nanoparticles. Only 18% of the selected papers are related to the very broad area of life sciences, mostly focusing on nanoparticle toxicology. In Figure 1, we can also see how the distribution of these papers’ topics evolves over time. We can see that from about 2007 there is a large growth in fields other than traditional dimensional metrology, progressing towards nanomaterials and life sciences, and such papers even seem to have much larger citation potential. Looking at the collected information, one would guess that before 2007 a typical “nanometrologist” worked at a national metrology institute and was dealing with Scanning Probe Microscopy. This is no longer the case, even if there are still a number of scientists working in this area, including the author of this editorial.

So, were the expectations about the growth of nanometrology found in the old review papers met? From the above survey, we could conclude that they were met only partially. The yearly number of papers related to nanometrology is stagnating at a value of around 70 papers/year since 2005. Does this mean that nanoscale measurement research has not evolved? Most likely not. If we search for publications related to the term “nanoscale” together with the term “measurement science”, we see that yearly publication counts for both topics are constantly growing. In addition, when going through the selected papers discussed above, we can see that many existing and growing areas are under-represented—there are only a few papers about various electron microscopy techniques, not many papers about analytical techniques and no papers about super-resolution methods in biology. Such methods are certainly “measurement science at nanoscale”, and there is certainly a continuous development in them; however, the authors for some reason do not call such work “nanometrology”. This might sound like only a minor terminological difference, but it might also partly reflect gaps that exist in reality. The connection of national metrology institutes to the life sciences or to academia is, unfortunately, sometimes weaker than to industry, and we can often observe that people are talking different languages. So, even if the whole field of nanoscale measurement science is growing, it is important to constantly search for new interconnections between academia, industry and metrology institutes, raising the aspects of metrological traceability in the nanoscale world. The increasing variability in paper topics that we can see in Figure 1 gives us hope that this is already happening.

In the past few years, nanometrology began facing new challenges, and many more are appearing even now. For example, projects related to the re-definition of the Si units were also targeting nanoscale measurements, either directly, e.g., by improving methods for silicon sphere surface and thin film measurements, or indirectly, e.g., by studying parameters such as silicon lattice spacing. This had led to a novel traceability route for nanometrology based on silicon lattice parameters [3], namely, suitable for Transmission Electron Microscopy and Scanning Probe Microscopy. This is one of the most visible direct impacts of the redefinition in the area of nanoscale measurement science. An example of a present challenge is establishing measurements for the emerging quantum technologies, which can bring to metrology novel types of high-precision metrology standards but will also increase the need for the characterization of various physical properties at the nanoscale [4].

As can be seen, the area of nanoscale measurements is very diverse, and as it is growing, it is becoming much broader than what the people who coined the term nanometrology could have anticipated. We are therefore happy that, in this Special Issue, we can present papers from various fields, coming from both academia and metrology institutes. This includes the aspects of traceability for analytical techniques, in particular X-ray-based techniques [5], electrical SPM [6,7], quantitative data processing [8], tools for estimation related to data processing [9] and exploring novel routes for quantitative measurements [10]. We hope that this will provide a nice example of many different flavors of today’s nanometrology. 

## Figures and Tables

**Figure 1:**
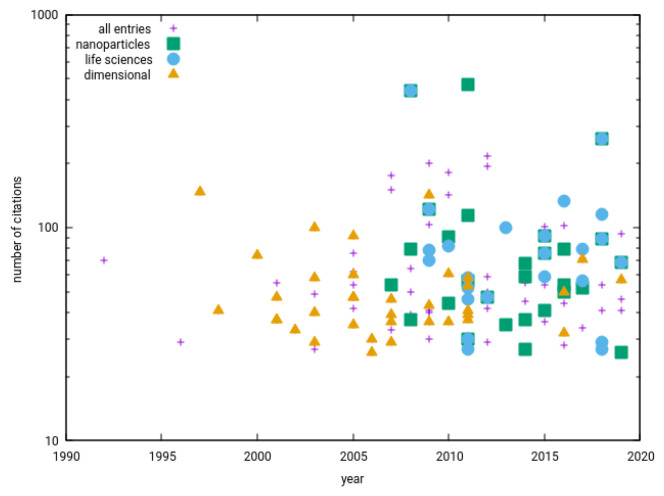
Temporal evolution of topics of the most cited 10% of papers with the subject “nanometrology”.
